# Patterns of ZMC and Le Fort Fractures under the Impact of the COVID-19 Pandemic—“A Changing Face?”

**DOI:** 10.3390/jcm13164662

**Published:** 2024-08-08

**Authors:** Florian Dudde, Johannes Schunk, Thomas Telschow, Filip Barbarewicz, Oliver Schuck, Manfred Giese, Wilken Bergmann

**Affiliations:** Department of Oral and Maxillofacial Surgery, Army Hospital Hamburg, 22049 Hamburg, Germany

**Keywords:** pandemic, midface fractures, patterns, distribution, COVID-19

## Abstract

**Background**: The aim of this study was to analyze the impact of the COVID-19 pandemic on midfacial fracture patterns/distributions and circumstances in a German craniomaxillofacial trauma center. **Methods**: This retrospective study compared the midface fracture patterns (excluding nasal fractures) of patients in the pre-COVID (PC) era (February 2019–January 2020) with patients in the intra-COVID (IC) era (February 2020–January 2021). In addition to baseline characteristics, the type of midface fractures, the circumstances leading to midface fractures, and hospital admissions/treatments were analyzed. **Results**: During the COVID-19 pandemic, a reduction in the total number of midface fractures was observed (PC = 88 vs. IC = 57). No significant differences were found regarding the midfacial fracture localization between both periods. During the pandemic, there was a significant increase in falls, accidents at home, and virus/flu-associated syncopes. At the same time, a significant decrease in sports accidents, interpersonal violence, and alcohol-related accidents leading to midface fractures was recorded. Furthermore, there was a significant increase in accidents during the morning time with a simultaneous reduction in accidents during the nighttime. In addition to that, a significant delay in days from trauma leading to midface fracture until hospital admission and surgical treatment (ORIF) was revealed. **Conclusions**: Despite the limitations of a monocentric retrospective study, the current findings lead to the conclusion that the COVID-19 pandemic had a significant impact on the patterns and circumstances leading to midface fractures. Analyzing the specific characteristics of patients suffering from midfacial fractures under the influence of the COVID-19 period can represent added value in order to treat facial fractures in future pandemics.

## 1. Introduction

Fractures of the facial skull are among the most common fracture locations [1]. There are different fracture types and classifications, particularly in the midface area. In principle, a distinction can be made between central, centrolateral, and lateral midface fractures [2,3]. In the area of the central/centrolateral midface, Le Fort fractures (Le Fort I–III) and nasal bone fractures represent the typical fracture types [4,5]. In centrolateral and lateral fractures, the zygomatico–maxillary complex (ZMC) is the most common fracture location [6]. Furthermore, adjacent anatomical structures such as the zygomatic arch (isolated zygomatic arch = IZA) or the orbital floor (isolated orbital floor = IOF) can also fracture with/without the other midface fractures [7,8]. Sports accidents, interpersonal violence, falls, and traffic accidents are among the most common causes of midface trauma [9].

Symptoms of central midfacial fractures include occlusion disorders (i.e., Le Fort I), pain, hematoma, and/or bleeding from the mouth and nose [10]. If the orbit is involved (infraorbital rim, orbital floor), bony step formation with crepitations can sometimes be palpable, as well as a slight sinking of the orbital bulbus with accompanying monocular hematoma [11,12]. Depending on the extent of the fracture, patients may also complain of diplopia as well as paresthesia of the infraorbital nerve [10]. Furthermore, displaced ZMC/IZA fractures can lead to impairment of mouth opening/closing [10]. A feared complication of infraorbital or periorbital trauma is the occurrence of a retrobulbar hematoma with the risk of permanent damage to the optic nerve and subsequent blindness in the affected eye [13]. In addition to clinical examination, imaging procedures play a crucial role in the diagnosis of midface trauma, particularly regarding the decision for conservative or surgical treatment [14]. Computed tomography (CT) is the imaging method of choice, particularly in the context of acute trauma diagnostics [15]. In particular, displaced midface fractures with accompanying clinical symptoms (i.e., double vision, paresthesia, and occlusion disorders) are treated surgically (ORIF).

The COVID-19 pandemic presented the global healthcare system with immense challenges and, due to capacity shortness, led to several adjustments, particularly in acute trauma and emergency care [16,17]. The rapid spread and high infectivity of the pandemic caused by the SarsCoV-2 virus led to a drastic reduction in elective surgical procedures with the aim of maintaining the surgical capacity for emergency and trauma care and at the same time securing intensive care capacities throughout the treatment of COVID-19 infections [18,19]. Furthermore, country-wide temporary lockdowns were introduced in many countries with the aim of preventing the rapid spread of this infectious disease [20]. This, as well as the wearing of personal protective equipment (i.e., FFP-2 masks) and the multiple vaccinations against COVID-19, affected the lives of people on a global level [21]. This was also noticeable in the changing circumstances leading to trauma and the changing numbers of trauma-related emergency presentations [22,23].

Since operative fracture treatment represents a significant cost factor in the healthcare system, an analysis of the frequencies and types of fractures is essential, especially given the lack of resources during a pandemic. It can be assumed that these healthcare costs may be reduced, and operative capacities may be used more efficiently by establishing preventive measures through the identification of specific fracture patterns within and without pandemic times.

However, the fracture patterns and treatment challenges/changes in midface fractures during the pandemic in Germany have not been studied yet. Therefore, the aim of this study is to investigate the impact of the COVID-19 pandemic on the Le Fort, ZMC, IOFm, and IZA fracture distributions; the fracture circumstances/patterns; and treatment modalities and to compare these results with the pre-COVID data (2019) in a craniomaxillofacial trauma center in Hamburg. We hypothesized that there were significant changes in fracture patterns during the pandemic compared to the pre-pandemic era.

## 2. Materials and Methods

### 2.1. Data Collection

The present study retrospectively examined patients who presented to the Emergency Department of the Army Hospital Hamburg with ZMC, IOF, IZA, or Le Fort fractures between February 2019 and January 2021. Patients were divided by their date of admission into two cohorts (pre-COVID (PC) February 2019–January 2020 vs. intra-COVID (IC) February 2020–January 2021). All patients were at least 18 years of age and fully capable of providing consent to the procedure/diagnostics and suffered from at least one fracture of the above. Exclusion criteria were incomplete documentation, concomitant mandible fracture, and/or nasal bone fracture. A total of 145 patients were included in this retrospective study.

### 2.2. Baseline Characteristics and Midface Fracture Patterns

Baseline characteristics, such as gender and age, were retrospectively identified for every patient from the patients’ digital file (Nexus AG, Donaueschingen, Germany) and anonymized. Ethical approval was waived by the clinical ethics committee of the hospital. Furthermore, the midface fracture patterns (fracture type, dental trauma, soft tissue injury, and conservative/operational treatment) were identified for every patient. 

### 2.3. Circumstances of Fractures

In addition, the circumstances leading to midface fractures were analyzed for every patient (fall, road traffic accident, sports accident, interpersonal violence, domestic violence, home accident, alcohol-related accident, work/free-time related accident, month, and time of day).

### 2.4. Changes in Hospital Admission/Hospital Circumstances

Furthermore, the time difference (days) from trauma until hospital admission, length of stay in hospital, and days from trauma until operation were retrospectively identified for all patients.

### 2.5. Statistical Analysis

Descriptive analysis was used to display patients’ baseline characteristics. Normally distributed continuous variables are presented as mean ± standard deviation and binary variables are presented as absolute and relative frequencies. The comparison of continuous variables was performed by Student’s *t*-test. A chi-square test was used for the analysis of binary variables. A *p*-value < 0.05 was considered statistically significant. All statistical analyses were performed using the SPSS version 28.0 statistical package (IBM, Markham, ON, Canada). 

## 3. Results

### 3.1. Baseline

A total of 145 patients were included in this study, comprising 90 male patients and 55 female patients (Table 1). The mean age of the patients was 55.87 years (Table 1). Patients were divided into two cohorts (pre-COVID: *n* = 88 vs. intra-COVID: *n* = 57) based on their date of admission.

### 3.2. Fracture Patterns

**Figure 1:**
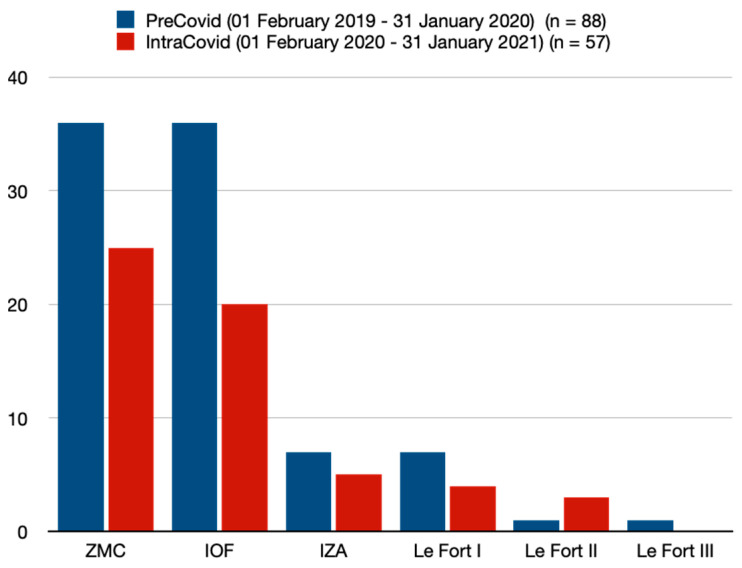
Type of fracture. ZMC = zygomatico–maxillary complex fracture; IOF = isolated orbital floor fracture; IZA = isolated zygomatic arch fracture.

In the PC cohort, a total of 88 patients presented with ZMC, IOF, IZA, or Le Fort fractures. In the PC period, ZMC fractures (40.9%) and IOF fractures (40.9%) were the most common fracture types (Table 2, Figure 1). Notably, 8.0% of all patients in the PC cohort presented with an IZA fracture (Table 2, Figure 1). A total of seven patients (8.0) in the PC period suffered from a Le Fort I fracture (Table 2, Figure 1). One patient each suffered Le Fort II and Le Fort III fractures in the PC cohort (Table 2, Figure 1). In the IC period, the ZMC fracture was the most common fracture type, at 43.9% (Table 2, Figure 1). The percentage of IOF fractures decreased to 35.1% (Table 2, Figure 1). There was a slight increase in IZA fractures (8.8%) and Le Fort II fractures (5.3%) in the IC period (Table 2, Figure 1). The number of Le Fort I fractures (7.0%) and Le Fort III fractures (0%) decreased in the IC period (Table 2, Figure 1). There were no significant differences between the two cohorts regarding concomitant soft tissue injuries (PC = 55.7% vs. IC = 50.9%) (Table 2). There were no significant differences between the two periods focusing on concomitant tooth loss, tooth avulsion, tooth extrusion, tooth intrusion, or tooth fracture (Table 2). A sharp decrease from 61.4% (PC) to 47.4% (IC) was observed in the number of surgical interventions/treatments (ORIF) in all fractures (*p* = 0.097) (Table 2).

### 3.3. Circumstances of Fractures

With regard to the circumstances leading to midface fractures, significant differences were found between both cohorts (Table 3). There was a highly significant increase in falls from 43.2% (PC) to 66.7% (IC) (*p* = 0.006) (Table 3). Furthermore, there was a significant decrease in interpersonal violence from 33.0% (PC) to 14.0% (IC) (*p* = 0.011) (Table 3). Regarding the rates of domestic violence, there was an increase of 6.0% in the IC period (Table 3). The number of alcohol-related midface fractures decreased from 30.7% (PC) to 10.5% (IC) (*p* = 0.005) (Table 3). In addition, there was a significant decrease in sports accidents leading to midface trauma, from 19.3% (PC) to 7.0% (IC) (*p* = 0.040) (Table 3). The number of virus/flu-associated syncopes increased significantly from 2.3% (PC) to 17.5% (IC) (*p* = 0.001) (Table 3). However, the rates of road traffic accidents (car, bicycle, e-scooter, motorcycle, and walking) did not show any differences between both periods (Table 3). Remarkably, there was a highly significant increase in accidents at home leading to midface fractures, from 23.9% (PC) to 56.1% (IC) (*p* = < 0.001) (Table 3).

There were no significant differences regarding the monthly distribution of the midface fractures described above (Table 3). In the PC cohort, most fractures occurred in October (15.9%) and May (10.2%) (Table 3). The lowest numbers of midface fractures in the PC cohort were found in June (5.7%) (Table 3). In the IC cohort, most fractures occurred in March (14.0%), July (12.3%), and September (12.3%) (Table 3). October was the month with the lowest number of midface fractures in the IC cohort (0%) (Table 3).

**Figure 2:**
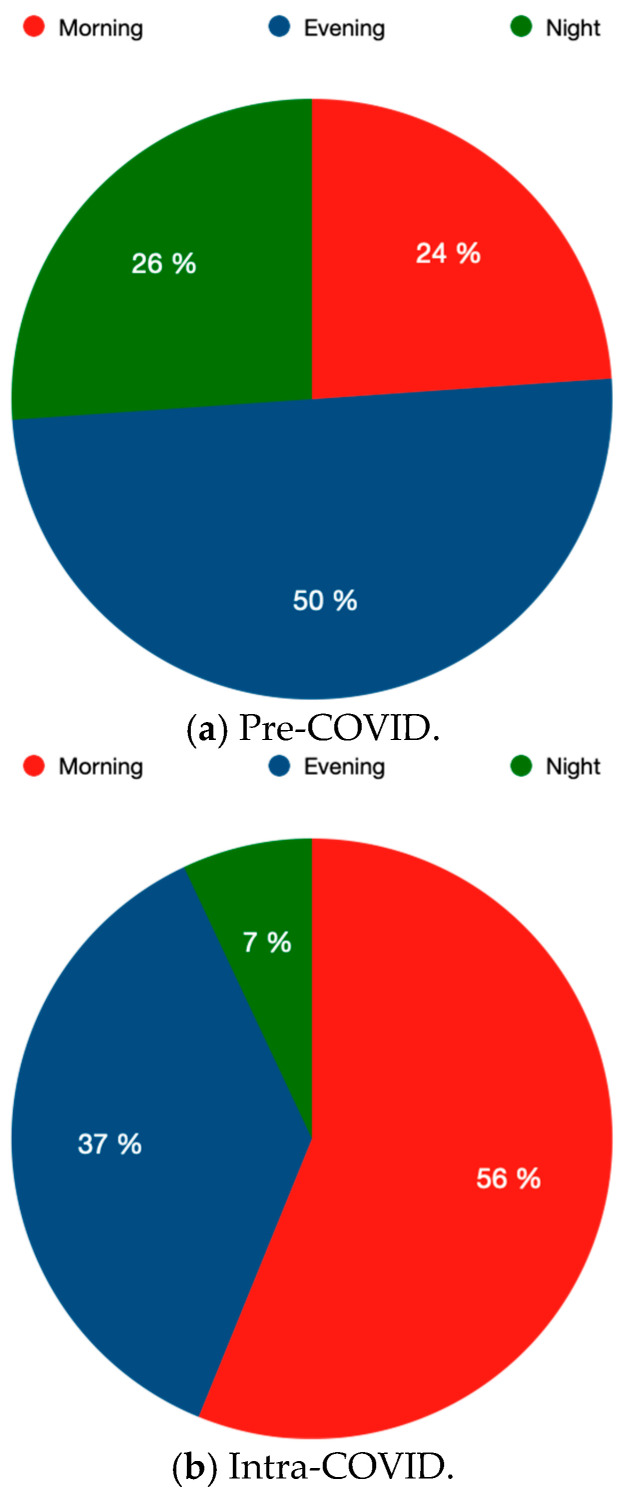
Changes in the time of trauma occurrence considering pre-COVID vs. intra-COVID: morning = 8 a.m.–4 p.m.; evening = 4 p.m.–12 p.m., night = 12 a.m.–8 a.m.

Furthermore, there were significant differences between the two cohorts regarding midface fractures on weekdays or weekends (Table 3). There was a significant decrease in midface fractures on the weekends (PC = 56.8% vs. IC = 36.8%; *p* = 0.019) (Table 3). The time of accidents leading to midface fractures also showed significant differences between the two periods (Table 3, Figure 2). There was a highly significant increase in midface fractures occurring in the morning (PC = 23.9% vs. IC = 56.1%; *p* = < 0.001) (Table 3, Figure 2). In addition to that, there was a highly significant decrease in midface fractures occurring in the nighttime (PC = 26.1% vs. IC = 7.0%; *p* = 0.004) (Table 3, Figure 2). Furthermore, a decrease in midface fractures occurring in the evening was observed (PC = 50.0% vs. IC = 36.8%; *p* = 0.120) (Table 3, Figure 2). An increase in work-related midface fractures from 8.0% (PC) to 14.0% (IC) (*p* = 0.240) was also observed (Table 3).

### 3.4. Changes in Hospital Circumstances

Significant differences were recorded regarding the midface fracture/trauma circumstances (Table 4). There was a significant increase in the days from trauma leading to midface fracture until presentation at our department (PC = 1.25 days vs. IC = 2.19 days; *p* = 0.029) (Table 4). The days from trauma to surgery (ORIF) also showed a significant increase in the IC cohort (PC = 1.42 days vs. IC = 2.56 days; *p* = 0.016) (Table 4). The mean length of stay in the hospital decreased from 4.63 days (PC) to 4.28 days (IC) without significant differences (Table 4).

## 4. Discussion

The aim of the present study was to investigate the influence of the COVID-19 pandemic on the patterns/circumstances of midface fractures. The present study showed a significant reduction in the total number of midface fractures from 88 (PC) to 57 (IC). Comparable studies also demonstrated a significant reduction in the number of maxillofacial fractures during the COVID-19 pandemic [24,25].

With regard to the distribution/location of midface fractures, there were no significant differences between the two periods in the present study. At approximately 40%, ZMC fractures were the most common midface fractures in both cohorts and correspond with the findings of comparable studies [24,26].

Interestingly, there was an increase in patients’ age in the PC cohort by approximately 5 years. This corresponds with the results of the studies by Salzano et al. and Meisgeier et al. in which a significant increase was found in midface fractures among elderly patients under the influence of the COVID-19 pandemic [24,27].

Regarding the circumstances leading to midface fractures, a significant increase in falls as well as a simultaneous decrease in interpersonal violence was noted during the pandemic. These findings correspond with the results from comparable studies from Australia and India [28,29]. Furthermore, in the present study, a significant increase in virus/flu-associated syncopes leading to midface fractures was found during the IC period. A similar observation has already been described regarding mandible fractures under the impact of the COVID-19 pandemic [25].

The nationwide lockdowns led to a far-reaching impact on the everyday lives of people around the globe. This also led to a reduction in physical activities in the spirit of “social distancing” [30]. Consecutively, the present study and comparable studies showed a significant reduction in sports accidents leading to (mid)facial trauma [25,31].

Along with the lockdowns, people spent more time at home both privately and workwise (i.e., home office) [32]. Consecutively, the present study revealed a significant increase in accidents at home leading to midface fractures.

In addition to that, the present study showed a significant increase in accidents leading to midface fractures during weekdays with a simultaneous decrease in accidents during the weekends. A similar trend was already evident for the occurrence of mandible fractures under the influence of the COVID-19 pandemic [25]. A possible explanation exists in the effects of the nationwide lockdowns, which particularly restricted leisure activities on the weekends. This is also accompanied by the significant decrease in alcohol-related accidents leading to midface fractures in the present study. The decrease in alcohol-related facial trauma was also observed in the study by Boom et al. [33].

Furthermore, the present study showed significant changes with regard to the time of day of accidents leading to midfacial fractures. There was a significant increase in accidents during the morning time leading to midfacial fractures, while at the same time, a significant decrease in accidents during the nighttime was noted. In accordance with the reasonable explanation already mentioned, the effects of the nationwide lockdowns, which particularly restricted/made nighttime activities impossible, also influenced the time of trauma leading to facial fractures [25].

The effects of the COVID-19 pandemic on hospital capacities and admissions were also shown in the present study. There was a significant increase in days from trauma leading to midface fracture until emergency presentation/admission in the IC period. The time delay (hospital admission) has also been demonstrated in other medical specialties [34,35]. In the present study, a significant increase in days from initial trauma leading to midface fracture until surgery (ORIF) was also observed. The limited surgical capacities during the COVID-19 pandemic were also noticeable in the treatment of midface fractures. Furthermore, the delay until surgery was also evident outside the field of (cranio)maxillofacial surgery [36,37]. In addition to that, the results showed an increase in conservative treatment during the pandemic, which can also be linked to the oversaturation of the healthcare system and/or by the type of trauma, since falls may be less prone to need surgical treatment in relation to interpersonal violence or sports-related causes. 

The findings of the present study are limited to certain facts. This study is based on a single center’s experience using a retrospective study design with limited power. Although the number of study participants and patients with midface fractures is comparable to other craniomaxillofacial trauma centers, the data need to be analyzed in a larger context due to the fact that the numbers of midface fractures may differ throughout different regions and craniomaxillofacial trauma centers. In particular, prospective studies in pandemic settings may help to reduce the influence of confounders (i.e., age, sex, and alcohol). Therefore, the available data should be examined in the context of international multicenter studies with larger populations and midface traumata. Since operative fracture treatment represents a significant cost factor in the healthcare system, an analysis of the frequencies and types of fractures is essential, especially given the lack of resources during a pandemic. It can be assumed that these healthcare costs may be reduced, and operative capacities may be used more efficiently by establishing preventive measures in order to lower the pandemic-specific fracture patterns.

In summary, the COVID-19 pandemic had a significant impact on the patterns and circumstances leading to midface fractures. In addition to the reduction in the total number of midface fractures during the pandemic, there was a significant increase in falls, accidents at home, and virus/flu-associated syncopes in the present study. At the same time, a significant decrease in sports accidents, interpersonal violence, and alcohol-related fractures leading to midface fractures was recorded. Furthermore, there was a significant increase in accidents during the morning time with a simultaneous reduction in accidents during the nighttime. In addition to that, a significant delay in days from trauma leading to midface fracture until hospital admission and surgical treatment (ORIF) was revealed.

## Figures and Tables

**Table 1:** Baseline characteristics.

Variable	Pre-COVID (February 2019–January 2020) (*n* = 88)	Intra-COVID (February 2020–January 2021) (*n* = 57)	*p*-Value
Age (years)	53.73 ± 22.66	59.88 ± 24.57	0.125
Gender			0.629
Male	56 (63.6)	34 (59.6)	
Female	32 (36.4)	23 (40.4)	

Note: Data are presented as mean (SD) and/or absolute values (percentage).

**Table 2:** Fracture patterns.

Variable	Pre-COVID (February 2019–January 2020) (*n* = 88)	Intra-COVID (February 2020–January 2021) (*n* = 57)	*p*-Value
Concomitant soft tissue injury	49 (55.7)	29 (50.9)	0.571
Tooth loss	4 (4.5)	5 (8.8)	0.303
Tooth avulsion	1 (1.1)	0 (0)	0.419
Tooth extrusion	4 (4.5)	1 (1.8)	0.368
Tooth intrusion	3 (3.4)	1 (1.8)	0.552
Tooth fracture	5 (5.7)	5 (8.8)	0.473
Operation (ORIF)	54 (61.4)	27 (47.4)	0.097
Conservative treatment (MMF)	34 (38.6)	30 (52.6)	0.097
Fracture location			
ZMC Fracture	36 (40.9)	25 (43.9)	0.576
Isolated orbital floor fracture	36 (40.9)	20 (35.1)	0.323
Isolated zygomatic arch fracture	7 (8.0)	5 (8.8)	0.861
Le Fort I fracture	7 (8.0)	4 (7.0)	0.835
Le Fort II fracture	1 (1.1)	3 (5.3)	0.138
Le Fort III fracture	1 (1.1)	0 (0)	0.419

ZMC = zygomatico–maxillary complex fracture. Note: Data are presented as absolute values (percentage).

**Table 3:** Causes for fractures.

Variable	Pre-COVID (February 2019–January 2020) (*n* = 88)	Intra-COVID (February 2020–January 2021) (*n* = 57)	*p*-Value
Fall	38 (43.2)	38 (66.7)	**0.006**
Sports accident	17 (19.3)	4 (7.0)	**0.040**
Epileptic accident	2 (2.3)	0 (0)	0.431
Road Traffic accidents	15 (17.0)	10 (17.5)	0.938
Car	5 (5.7)	4 (7.0)	0.745
Bicycle	7 (8.0)	4 (7.0)	0.835
E-Scooter	3 (3.4)	2 (3.5)	0.974
Motorcycle	0 (0)	0 (0)	/
Walking	0 (0)	0 (0)	/
Interpersonal violence	29 (33.0)	8 (14.0)	**0.011**
Domestic violence	7 (8.0)	8 (14.0)	0.240
Accident at home	21 (23.9)	32 (56.1)	**<0.001**
Alcohol-related	27 (30.7)	6 (10.5)	**0.005**
Virus/Flu-associated Syncope	2 (2.3)	10 (17.5)	**0.001**
Month			0.136
January	4 (4.5)	5 (8.8)	
February	8 (9.1)	2 (3.5)	
March	8 (9.1)	8 (14.0)	
April	8 (9.1)	5 (8.8)	
May	9 (10.2)	6 (10.5)	
June	5 (5.7)	3 (5.3)	
July	6 (6.8)	7 (12.3)	
August	8 (9.1)	3 (5.3)	
September	6 (6.8)	7 (12.3)	
October	14 (15.9)	0 (0)	
November	6 (6.8)	6 (10.5)	
December	6 (6.8)	5 (8.8)	
Weekday	38 (43.2)	36 (63.2)	**0.019**
Weekend	50 (56.8)	21 (36.8)	**0.019**
Time of accident			
Morning	21 (23.9)	32 (56.1)	**<0.001**
Evening	44 (50.0)	21 (36.8)	0.120
Night	23 (26.1)	4 (7.0)	**0.004**
Work-related accident	7 (8.0)	8 (14.0)	0.240

Note: Data are presented as absolute values (percentage): morning = 8 a.m.–4 p.m.; evening = 4 p.m.–12 p.m., night = 12 a.m.–8 a.m. Significant *p*-values are presented in bold.

**Table 4:** Changes in hospital admission.

Variable	Pre-COVID (February 2019–January 2020) (*n* = 88)	Intra-COVID (February 2020–January 2021) (*n* = 57)	*p*-Value
Days from trauma to presentation	1.25 ± 1.84	2.19 ± 3.31	**0.029**
Days from trauma to operation	1.42 ± 1.66	2.56 ± 3.89	**0.016**
Days in hospital	4.63 ± 3.02	4.28 ± 3.51	0.531

Note: Data are presented as absolute values and percentages. Significant *p*-values are presented in bold.

## Data Availability

The data presented in this study are available on request from the corresponding author. The data are not publicly available due to privacy.
